# Association of Vascular Risk Factors and Genetic Factors With Penetrance of Variants Causing Monogenic Stroke

**DOI:** 10.1001/jamaneurol.2022.3832

**Published:** 2022-10-27

**Authors:** Bernard P. H. Cho, Eric L. Harshfield, Maha Al-Thani, Daniel J. Tozer, Steven Bell, Hugh S. Markus

**Affiliations:** 1 Stroke Research Group, Department of Clinical Neurosciences, University of Cambridge, Cambridge, United Kingdom

## Abstract

**Importance:**

It is uncertain whether typical variants causing monogenic stroke are associated with cerebrovascular disease in the general population and why the phenotype of these variants varies so widely.

**Objective:**

To determine the frequency of pathogenic variants in the 3 most common monogenic cerebral small vessel diseases (cSVD) and their associations with prevalent and incident stroke and dementia.

**Design, Setting, and Participants:**

This cohort study is a multicenter population-based study of data from UK Biobank participants recruited in 2006 through 2010, with the latest follow-up in September 2021. A total of 9.2 million individuals aged 40 to 69 years who lived in the United Kingdom were invited to join UK Biobank, of whom 5.5% participated in the baseline assessment. Participants eligible for our study (n = 454 756, excluding 48 569 with incomplete data) had whole-exome sequencing and available data pertaining to lacunar stroke-related diseases, namely stroke, dementia, migraine, and epilepsy.

**Exposures:**

*NOTCH3, HTRA1*, and *COL4A1/2* pathogenic variants in monogenic stroke; Framingham cardiovascular risk; and ischemic stroke polygenic risk.

**Main Outcomes and Measures:**

Primary outcomes were prevalent and incident stroke and dementia. Odds ratios (ORs) and hazard ratios (HRs) were adjusted for age, sex, ethnicity, exome sequencing batch, and top 10 genetic principal components.

**Results:**

Of the 454 756 participants (208 027 [45.8%] men; mean [SD] age, 56.5 [8.1] years), 973 participants carried *NOTCH3* variants, 546 carried *HTRA1* variants, and 336 carried *COL4A1/2* variants. Variant carriers were at least 66% more likely to have had stroke. *NOTCH3* carriers had increased vascular dementia risk (OR, 5.42; 95% CI, 3.11-8.74), *HTRA1* carriers an increased all-cause dementia risk (OR, 2.17; 95% CI, 1.28-3.41), and *COL4A1/2* carriers an increased intracerebral hemorrhage risk (OR, 3.56; 95% CI, 1.34-7.53). *NOTCH3* variants were associated with incident ischemic stroke and vascular dementia. *NOTCH3* and *HTRA1* variants were associated with magnetic resonance imaging markers of cSVD. Cardiovascular risk burden was associated with increased stroke risk in *NOTCH3* and *HTRA1* carriers. Variant location was also associated with risk.

**Conclusions and Relevance:**

In this cohort study, pathogenic variants associated with rare monogenic stroke were more common than expected in the general population and associated with stroke and dementia. Cardiovascular risk burden is associated with the penetrance of such variants. Our results support the hypothesis that cardiovascular risk factor control may improve disease prognosis in individuals with monogenic cSVD variants. This lays the foundation for future studies to evaluate the effect of early identification before symptom onset on mitigating stroke and dementia risk.

## Key Points

**Question:**

What factors are associated with penetrance of variants in monogenic cerebral small vessel disease (cSVD)?

**Findings:**

In this population-based cohort study of 454 756 individuals, *NOTCH3*, *HTRA1*, and *COL4A1/2* variants causing monogenic cSVD were associated with increased stroke and dementia risk. Cardiovascular risk factors were found to be associated with penetrance of these variants.

**Meaning:**

The results of this cohort study support the hypotheses that cardiovascular risk factor control may improve disease prognosis in individuals with monogenic cSVD variants and that identifying individuals early in life before symptom onset may help reduce stroke and dementia risk.

## Teaser

This cohort study examines data from UK Biobank participants who had whole-exome sequencing to determine the frequency of pathogenic variants in the 3 most common monogenic cerebral small vessel diseases and their associations with prevalent and incident stroke and dementia.

## Introduction

Several monogenic disorders are associated with stroke and vascular dementia. These predominantly cause lacunar stroke. The most common of these, cerebral autosomal dominant arteriopathy with subcortical infarcts and leukoencephalopathy (CADASIL), is caused by *NOTCH3* variants, and the second most frequent, CADASIL2, by autosomal dominant *HTRA1* variants.^1^ *COL4A1/2* variants can cause small vessel stroke and intracerebral hemorrhage.^2^

Monogenic stroke was thought to be rare, with an estimated prevalence of CADASIL of 4 per 100 000 in the United Kingdom.^3^ However, recent studies have reported pathogenic variants are more prevalent in the general population,^4^ with typical *NOTCH3* variants present in 1 in 452 individuals^5^ and *HTRA1* variants in 1 in 275.^6^

These findings raise 2 important questions. First, are these apparently “asymptomatic” variants associated with stroke and dementia? Second, why do some individuals who have these variants present with severe early-onset stroke and dementia while others remain asymptomatic?

Previously, it was thought that CADASIL was a disease with high penetrance and that most individuals with typical variants would experience early-onset stroke. More recently, the clinical phenotype of CADASIL has been shown to be widely variable, with some having a stroke young while others remaining stroke-free until their 80s.^7^ Several factors have been suggested to modulate phenotype, including variant location (variants in epidermal growth factor–like repeats [EGFRs] 1-6 are associated with more severe disease),^4^ the presence of cardiovascular risk factors,^8^ and modifying genes.^9^ The UK Biobank resource includes data on vascular risk factors and imputed genome-wide genotyping, allowing cardiovascular risk factor and polygenic risk scores to be calculated to investigate the importance of such modifying factors.

To explore this further, we analyzed the latest exome sequencing data from UK Biobank. We identified *NOTCH3*, *HTRA1*, and *COL4A1/*2 pathogenic variants that cause the 3 most common monogenic cerebral small vessel diseases (cSVDs). We determined the frequency of such variants and their associations with both prevalent and incident stroke and dementia. We also determined associations with magnetic resonance imaging (MRI) markers of cSVD.

## Methods

### Study Population

UK Biobank is a prospective study of more than 500 000 participants aged 40 to 69 years recruited across the United Kingdom in 2006 to 2010.^10^ Phenotypic data were collected through questionnaires and physical examinations. A subset of 100 000 individuals, selected based on traveling distance from the imaging center, underwent MRI. All MRIs were performed on Siemens Skyra 3.0-T scanners with identical acquisition parameters and quality control.^11^ In October 2021, whole-exome sequences of 454 756 participants were released and were assessed for this study. UK Biobank received ethical approval from the National Health Service National Research Ethics Service Northwest (21/NW/0157). Written informed consent was obtained from all participants. This study followed the Strengthening the Reporting of Observational Studies in Epidemiology (STROBE) reporting guideline.^12^

### Ascertainment of Pathogenic Variants

Variants in *NOTCH3* (chr19:15,159,038-15,200,995, GRCh38), *HTRA1* (chr10:122,458,551-122,514,907), *COL4A1* (chr13:110,148,963-110,307,157), and *COL4A2* (chr13:110,305,812-110,513,209) were extracted in PLINK format. The extracted variants were annotated using the Ensembl Variant Effect Predictor^13^ and then filtered by a priori pathogenicity criteria for each gene (as specified below).

For *NOTCH3*, we identified CADASIL variants that cause the gain or loss of cysteine in 1 of the 34 EGFR domains of the NOTCH3 protein (amino acid position 40-1373).^14,15^

*HTRA1* and *COL4A1/2* pathogenic variants are not stereotyped in the same way as *NOTCH3* variants. Therefore, we performed a systematic review to identify those that had been reported in patients with familial cSVD. We searched PubMed using the terms “(CARASIL OR HTRA1 mutation*) AND (COL4A1 mutation* OR COL4A2 mutation* OR COL4A1/2 mutation*)” and selected English publications up to February 6, 2022. These variants were classified using the American College of Medical Genetics and Genomics (ACMG) criteria^16^ and the Association for Clinical Genomic Science Best Practice Guidelines for Variant Classification in Rare Disease 2020.^17^ Only variants classified as pathogenic or likely pathogenic were included in subsequent analyses.

### Phenotypic Data Fields

History of vascular risk factors needed to calculate the Framingham cardiovascular risk score (FRS)^18^ and parental history of stroke were recorded. History of diseases, including migraine, migraine with aura, any stroke, ischemic stroke, intracerebral hemorrhage, vascular dementia, all-cause dementia, and epilepsy, were determined from self-report and hospital and death records (eTable 1 in the Supplement). Diagnoses of stroke, ischemic stroke, intracerebral hemorrhage, and vascular dementia after recruitment to UK Biobank were identified as incident cases.

### Brain Imaging Analysis

In the 38 332 participants with both exome sequences and MRI, measures were compared between participants harboring pathogenic variants and controls. Among the sequences acquired were T1-weighted, fluid-attenuated inversion recovery (FLAIR), and diffusion tensor imaging (DTI) images (full acquisition details previously reported,^19^ brief details provided in eTable 2 in the Supplement). We used measures for brain and white matter hyperintensities (WMH) volume generated by UK Biobank.^11^ Brain volume was estimated from T1-weighted images by SIENAX^20^ and normalized for head size. The WMH were quantified on FLAIR images through the brain intensity abnormality classification algorithm^21^ and normalized for brain volume. Degree of white matter ultrastructural damage was calculated using software to derive peak width skeletonized mean diffusivity (PSMD),^22^ which provides a summary measure from DTI. Additionally, DTI was used to derive structural brain networks via tractography analysis,^23^ from which global and local structural efficiency measures^24^ were derived in house.^25^ Following tractography, an anatomic labeling atlas^26^ was used to generate a connectivity matrix (detailed in Shen et al^27^). Global efficiency is estimated by averaging the number of steps it takes to go from any given node to any other; local efficiency is calculated from the efficiency of the connections between a given node and those nodes connected to it. Global network efficiency has been shown to be sensitive to damage in cSVD, correlate with cognitive impairment,^28^ and predict future dementia risk.^29^

### Calculation of Framingham Cardiovascular and Polygenic Risk Scores

To compare ischemic stroke risk associated with the *NOTCH3* and *HTRA1* variants and that of cardiovascular risk factors and common genetic variants, we calculated the FRS^18^ and an ischemic stroke polygenic risk score (PRS),^30^ respectively.

### Statistical Analysis

The effects of *NOTCH3*, *HTRA1*, and *COL4A1/2* variants on phenotypes were assessed by linear regression for continuous outcomes and logistic regression for binary outcomes. All the variant carriers only had 1 of the prespecified variants in these genes. A Firth correction was applied to logistic regression to reduce rare event bias.^31^ All regression models were adjusted for age, sex, ethnicity, exome sequencing batch, and the first 10 principal components of genetic ancestry. The WMH volumes were natural log-transformed for analyses.

Kaplan-Meier analyses and Cox proportional-hazards regression with Firth corrections (using time since recruitment to UK Biobank as the underlying timescale and adjusting for FRS, PRS, and the same covariates included in the above-mentioned regression analyses) were performed to compare the cumulative probability of incident disease in variant groups and in different PRS and FRS tiers. Assumptions of Cox regression were tested based on Schoenfeld residuals, and no violations were observed. When calculating hazard ratios (HRs) for ischemic stroke associated with 1-SD higher FRS and PRS as well as *NOTCH3* and *HTRA1* variant status, Cox proportional-hazards regression models were limited to participants without history of stroke, coronary heart disease, peripheral vascular disease, or congestive heart failure at recruitment. By dividing the HR associated with the variant status by that associated with 1-SD higher FRS or PRS (on the log scale, assuming a linear association), we estimated the increment of the FRS and PRS in SD that was predicted to be equivalent to the risk associated with the *NOTCH3* and *HTRA1* variants.^32^

We performed statistical tests for interaction between the FRS or PRS and variant status of each gene; for this, we divided the participants into 2 groups based on FRS or PRS: low (bottom 50%) and high (top 50%) risk.^33^ Multiplicative and additive interaction was assessed by analysis of variance and the synergy index, respectively.^34^ All statistical analyses were performed using R version 4.0.3 with 2-sided *P* values and *P* < .05 considered statistically significant.

## Results

### Systematic Review and Classification of the *HTRA1* and *COL4A1/2* Variants

After performing ACMG classification of *HTRA1* and *COL4A1/2* variants identified in the literature search (eFigure 1 in the Supplement), we found 63 pathogenic and likely pathogenic variants in *HTRA1*, 131 in *COL4A1*, and 21 in *COL4A2*. Although the *HTRA1* p.Gln151Lys variant was classified as likely pathogenic, we excluded it from our analyses based on evidence indicating it does not affect protease activity.^6^

### Prevalence and Distribution of the *NOTCH3*, *HTRA1*, and *COL4A1/2* Pathogenic Variants

Of the 454 756 participants (208 027 [45.8%] men; mean [SD] age, 56.5 [8.1] years), 973 were heterozygous *NOTCH3* carriers (1 in 467). Ninety-nine unique *NOTCH3* variants were identified, of which 54 were previously reported on dbSNP (eFigure 2 and eTable 3 in the Supplement).^35^ Variants were predominantly in EGFRs 7 through 34; only 22 participants (2%) had a *NOTCH3* variant in EGFRs 1 through 6. The most common *NOTCH3* variants were p.Arg1231Cys and p.Cys1222Gly, found in 255 and 212 individuals, respectively.

For *HTRA1*, 546 heterozygous carriers were found (1 in 832); 18 unique variants were identified (eFigure 3 and eTable 4 in the Supplement). Two-thirds of the identified variants affected the protease domain, and these were carried by 92% of *HTRA1* carriers. Notably, p.Arg227Trp was the most common *HTRA1* variant, found in 379 individuals.

For *COL4A1/2*, 336 heterozygous carriers were found (1 in 1353); 11 unique variants were identified (eFigures 4 and 5 and eTable 5 in the Supplement). All the identified variants affected the triple helix region. The most common variant in *COL4A1*/2 was p.Gly332Arg, found in 174 individuals.

### Association Between the *NOTCH3*, *HTRA1*, and *COL4A1/2* Variants and Prevalent Stroke, Vascular Dementia, and Other Clinical Features

**Figure 1.**
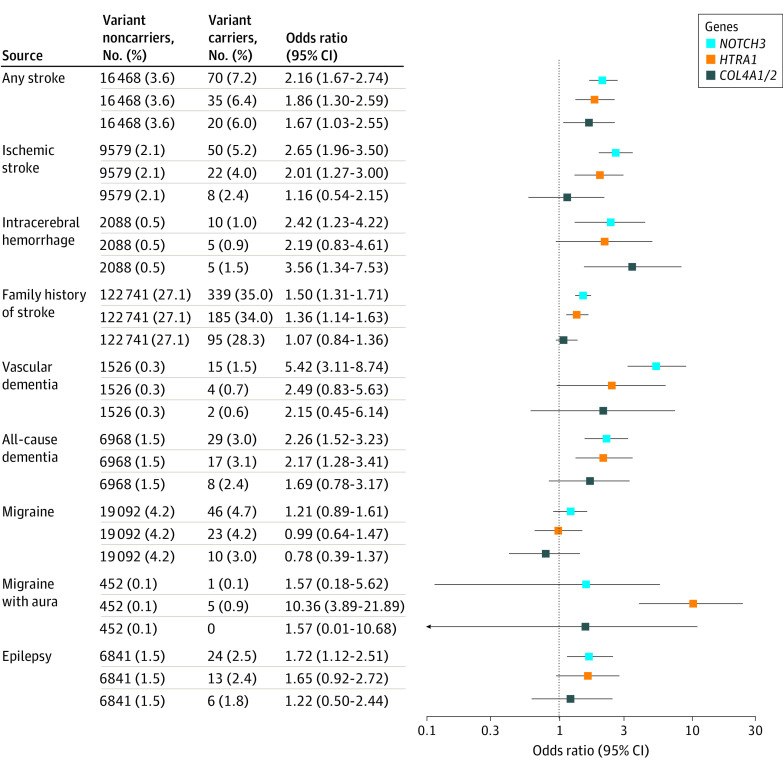
Association of the *NOTCH3*, *HTRA1*, and *COL4A1/2* Variants With Cerebral Small Vessel Disease–Related Diagnoses (N = 454 276) A Firth correction was applied to all the regression models, which were adjusted for age, sex, ethnicity, exome sequencing batch, and the first 10 genetic ancestry principal components. After accounting for multiple comparisons using a false-discovery rate of 5% with the Benjamini-Hochberg procedure, all the associations with the *NOTCH3* and *HTRA1* variants remained significant; only the associations with the *COL4A1/2* variants became insignificant (*P* value for any stroke risk changed from .04 to .18, and *P* value for intracerebral hemorrhage risk changed from .01 to .13).

The presence of a *NOTCH3* variant was associated with at least 2-fold higher odds of any stroke (odds ratio [OR], 2.16; 95% CI, 1.67-2.74; *P* = 3.2 × 10^−8^), ischemic stroke (OR, 2.65; 95% CI, 1.96-3.50, *P* = 5.9 × 10^−9^), and intracerebral hemorrhage (OR, 2.42; 95% CI, 1.23-4.22; *P* = .01). All-cause dementia (OR, 2.26; 95% CI, 1.52-3.23; *P* = .0001) and vascular dementia (OR, 5.42; 95% CI, 3.11-8.74; *P* = 2.7 × 10^−7^) were more prevalent in variant carriers. Population-attributable fractions showed that 0.21% of strokes and 0.77% of vascular dementias were attributed to *NOTCH3* variants. There was also an increased odds of epilepsy (OR, 1.72; 95% CI, 1.12-2.51; *P* = .01) and family history of stroke (OR, 1.50; 95% CI, 1.31-1.71; *P* = 6.0 × 10^−9^). No significant associations were found for migraine or migraine with aura (Figure 1).

The presence of an *HTRA1* variant was associated with increased risk of migraine with aura (OR, 10.36; 95% CI, 3.89-21.89; *P* = 7.6 × 10^−5^), any stroke (OR, 1.86; 95% CI, 1.30-2.59; *P* = .001), and ischemic stroke (OR, 2.01; 95% CI, 1.27-3.00; *P* = .004). All-cause dementia (OR, 2.17; 95% CI, 1.28-3.41; *P* = .005) was more prevalent in variant carriers. Among stroke and dementia cases, 0.09% of strokes and 0.14% of vascular dementias were attributed to *HTRA1* variants. Carriers were more likely to have a family history of stroke (OR, 1.36; 95% CI, 1.14-1.63; *P* = .001). No significant association was found with vascular dementia (OR, 2.49; 95% CI, 0.83-5.63; *P* = .10) or any other clinical outcomes (Figure 1).

The presence of a *COL4A1/2* variant was associated with increased risk of any stroke (OR, 1.67; 95% CI, 1.03-2.55; *P* = .04), which was accounted for by an increase in intracerebral hemorrhage risk (OR, 3.56; 95% CI, 1.34-7.53; *P* = .01), while there was no difference in ischemic stroke risk (OR, 1.16; 95% CI, 0.54-2.15; *P* = .69). Among strokes, 0.04% were attributed to the *COL4A1/2* variants (Figure 1).

After limiting our analyses to unrelated individuals with adjustment for FRS and PRS, variant carrier prevalence was consistent, and the disease associations, excluding the risk of any stroke associated with *COL4A1/2* variants, remained significant (eTables 6-8 in the Supplement).

### Association Between Variants and MRI Features of cSVD

Magnetic resonance images were available for 92 *NOTCH3*, 44 *HTRA1*, and 28 *COL4A1/2* carriers. *NOTCH3* variants were associated with increased WMH volume (standardized difference [β], 0.48; 95% CI, 0.32 to 0.64; *P* = 5.5 × 10^−9^) and white matter ultrastructural damage on PSMD (β, 0.64; 95% CI, 0.46 to 0.83; *P* = 8.5 × 10^−12^), as well as decreased local (β, −0.25; 95% CI, −0.45 to −0.05; *P* = .01) and global (β, −0.27; 95% CI, −0.47 to −0.07; *P* = .01) structural network efficiency (Table 1). They were associated with larger brain volume (β, 0.24; 95% CI, 0.07 to 0.40; *P* = .01).

**Table 1.** Standardized Effects of the *NOTCH3*, *HTRA1*, and *COL4A1/2* Variants on Different MRI Markers^a^

Markers	Variant noncarriers, No.	Variant carriers, No.	Standardized effect (95% CI)	*P* value
***NOTCH3***				
Brain volume	39 598	92	0.24 (0.07 to 0.40)	.01
WMH volume	38 295	89	0.48 (0.32 to 0.64)	<.001
PSMD	37 274	87	0.64 (0.46 to 0.83)	<.001
Local efficiency of structural brain network	34 486	84	−0.25 (−0.44 to −0.05)	.01
Global efficiency of structural brain network	34 486	84	−0.27 (−0.47 to −0.07)	.01
***HTRA1***				
Brain volume	39 405	44	0.21 (−0.03 to 0.45)	.08
WMH volume	38 110	42	0.55 (0.31 to 0.78)	<.001
PSMD	37 094	42	0.68 (0.41 to 0.95)	<.001
Local efficiency of structural brain network	34 313	42	−0.48 (−0.76 to −0.20)	<.001
Global efficiency of structural brain network	34 313	42	−0.50 (−0.77 to −0.22)	<.001
***COL4A1/2***				
Brain volume	39 598	28	0.14 (−0.16 to 0.44)	.35
WMH volume	38 295	23	−0.01 (−0.33 to 0.30)	.93
PSMD	37 274	24	0.06 (−0.29 to 0.41)	.73
Local efficiency of structural brain network	34 486	24	0.10 (−0.27 to 0.47)	.59
Global efficiency of structural brain network	34 486	24	0.11 (−0.25 to 0.48)	.55

Abbreviations: MRI, magnetic resonance imaging; PSMD, peak width skeletonized mean diffusivity; WMH, white matter hyperintensities.

^a^ Measurements of brain volume, WMH, and PSMD as well as local and global efficiency of structural brain network were analyzed with the presence of the variants through linear regression with adjustment for age at scan, ethnicity, sex, sequencing batch, and genetic principal components. The average local efficiency measures clustering/segregation and specialization within a network: that is, the capability to perform specialized processing within brain regions. Global efficiency concerns integration over the whole network and reflects the ability to rapidly combine information from different brain regions. Brain and WMH volumes were natural-log transformed.

*HTRA1* variants were associated with higher WMH volume (β, 0.55; 95% CI, 0.31 to 0.78; *P* = 5.6 × 10^−6^) and DTI-PSMD (β, 0.68; 95% CI, 0.42-0.95; *P* = 5.2 × 10^−7^) and lower local (β, −0.48; 95% CI, −0.76 to −0.20; *P* = .001) and global (β, −0.50; 95% CI, −0.77 to −0.22; *P* = .0004) structural efficiency (Table 1). They were not associated with brain volume. *COL4A1/2* variants were not associated with any of the MRI markers (Table 1).

### Association Between Variant Carriers and Incident Stroke and Vascular Dementia

**Figure 2.**
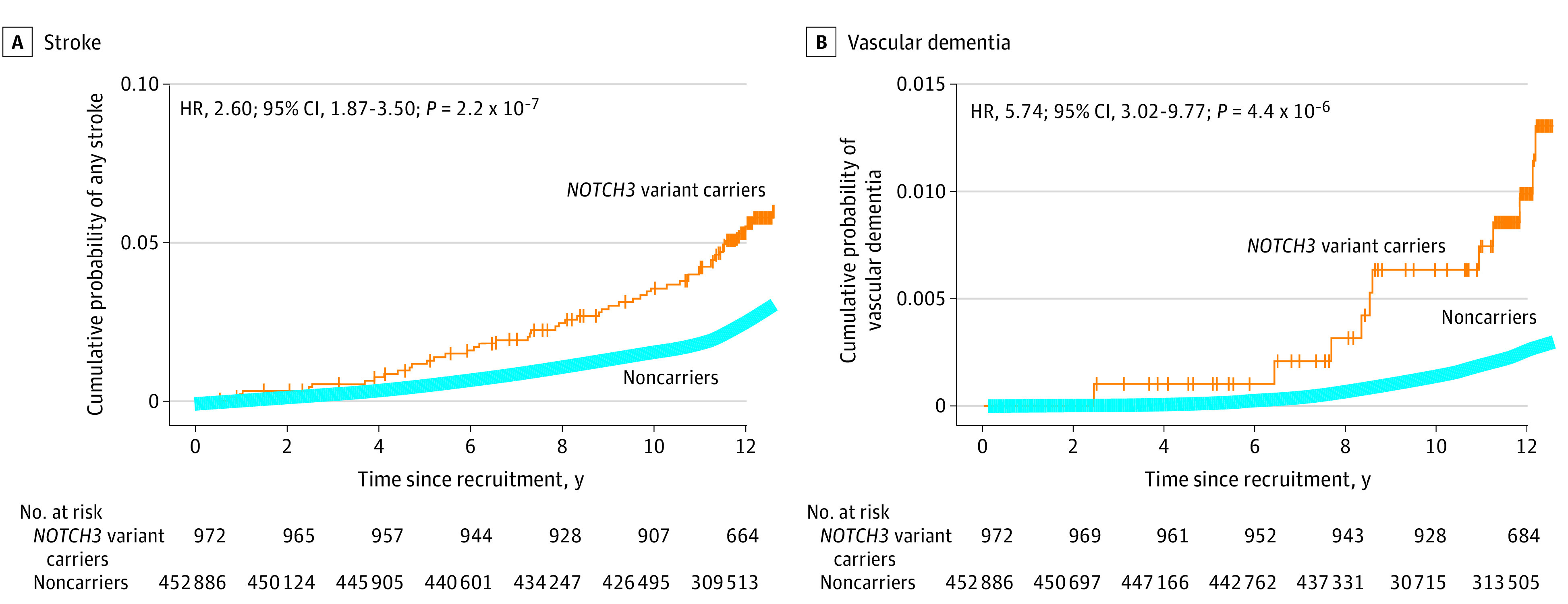
Survival of Stroke and Vascular Dementia Associated With *NOTCH3* Variant Carriers vs Noncarriers Hazard ratio (HR) was calculated through Cox regression with a Firth correction and adjustment for sex, ethnicity, exome sequencing batch, and the first 10 genetic ancestry principal components.

During follow-up for a median (IQR) duration of 12.6 years (11.8-13.2), *NOTCH3* variants were associated with incident stroke (HR, 2.60; 95% CI, 1.87-3.50; *P* = 2.2 × 10^−7^) and vascular dementia (HR, 5.74; 95% CI, 3.02-9.77; *P* = 4.4 × 10^−6^) (Figure 2). *HTRA1* variants were associated with incident stroke (HR, 1.80; 95% CI, 1.05-2.86; *P* = .03) but not with vascular dementia (HR, 3.08; 95% CI, 0.87-7.55; *P* = .08). For *COL4A1/2*, variant status was not predictive of incident stroke (HR, 1.03; 95% CI, 0.42-2.03; *P* = .95). None of the variant carriers had vascular dementia.

### Effect of Modulating Factors on Phenotype

#### Cardiovascular Risk Profile

We calculated the effect of vascular risk factors on ischemic stroke risk in individuals with and without variants. This analysis was limited to *NOTCH3* and *HTRA1* because significant associations with ischemic stroke had only been found for these variants.

**Figure 3.**
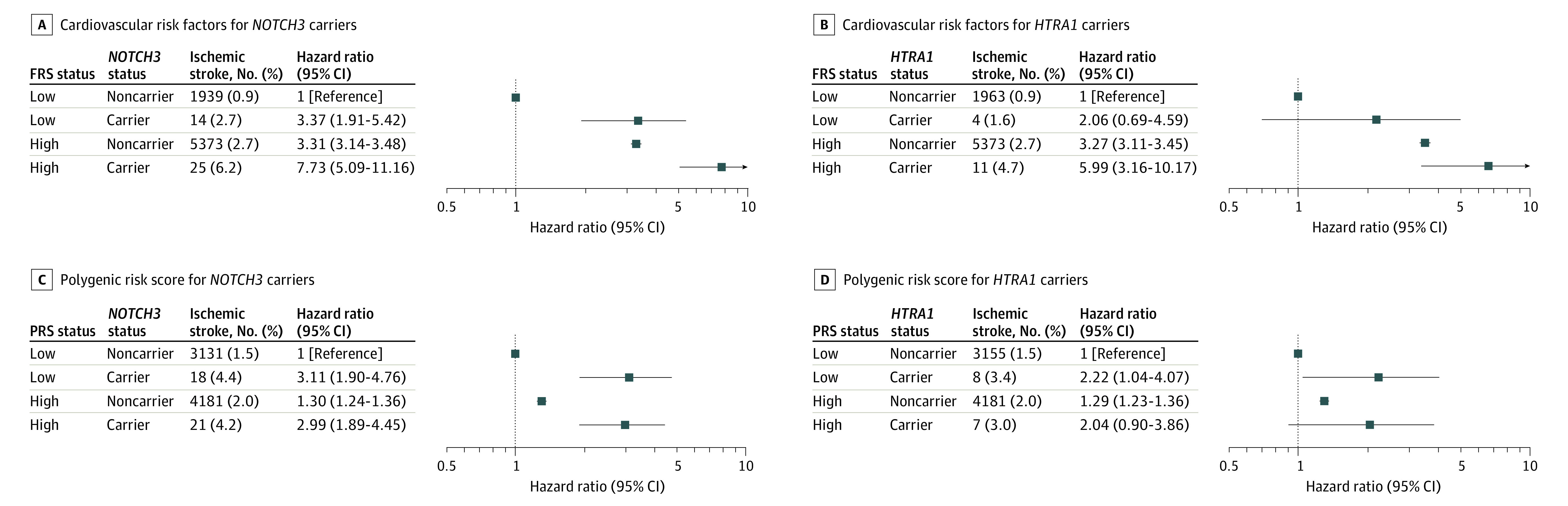
Interplay of Monogenic, Cardiovascular, and Polygenic Risk for Ischemic Stroke A, Risk of ischemic stroke by *NOTCH3* status and Framingham cardiovascular risk score (FRS) strata. For a multiplicative interaction with the categorical low and high FRS groupings, *P* = .07; for the additive interaction with categorical FRS, assessed using the synergy index, *P* < .001. B, Risk of ischemic stroke by *HTRA1* status and FRS strata. For a multiplicative interaction with the categorical FRS groupings, *P* = .85; for the additive interaction with categorical FRS, assessed using the synergy index, *P* < .001. C, Risk of ischemic stroke by *NOTCH3* status and polygenic risk score (PRS) strata. For a multiplicative interaction with the categorical low and high PRS groupings, *P* = .97; for the additive interaction with categorical PRS, assessed using the synergy index, *P* = .18. D, Risk of ischemic stroke by *HTRA1* status and PRS strata. For a multiplicative interaction with the categorical PRS groupings, *P* = .29; for the additive interaction with categorical PRS, assessed using the synergy index, *P* = .28.

Cardiovascular risk factor burden, as assessed by the FRS, increased ischemic stroke risk (HR for 1-SD higher FRS, 2.06; 95% CI, 2.01-2.12; *P* < .001). Having a higher FRS was associated with increased stroke risk in both variant carriers and noncarriers, although statistical significance was only seen in noncarriers (HR, 1.56; 95% CI, 1.47-1.65; *P* < .001) (Figure 3). No evidence for multiplicative interaction was observed. However, there was an additive interaction between FRS and *NOTCH3* and *HTRA1* carrier status (*NOTCH3* synergy index [SI], 1.66; 95% CI, 1.14-2.43; *P* < .001; *HTRA1 *SI, 1.60; 95% CI, 1.13-2.26; *P* < .001). We calculated that *NOTCH3* variants conferred the same risk as a 1.32-SD increase in FRS and *HTRA1* variants a 0.81-SD increase. These placed *NOTCH3* and *HTRA1* variant carriers as having a cardiovascular risk equivalent to the upper 9.3% and 20.9% of the population, respectively.

#### Polygenic Risk

Common variants, as assessed by PRS, increased stroke risk in all participants (HR for 1-SD higher PRS, 1.23; 95% CI, 1.20-1.26; *P* < .001). However, this appeared to affect only noncarriers of *NOTCH3* and *HTRA1* variants (Figure 3). Unlike the FRS, there was no additive interaction between PRS and *NOTCH3* and *HTRA1* carrier status (*NOTCH3* SI, 0.96; 95% CI, 0.48-1.94; *P* = .18; *HTRA1 *SI, 0.74; 95% CI, 0.25-2.25; *P* = .28). Also, additive interaction but not multiplicative interaction was found between the categorical FRS and PRS groupings (SI, 1.29; 95% CI, 1.24-1.34; *P* < .001). We calculated *NOTCH3* variants conferred the same risk as a 4.62-SD increase in PRS and *HTRA1* variants a 2.85-SD increase. *NOTCH3* and *HTRA1* variant carriers were associated with the same ischemic stroke risk as belonging to the upper 0.1% and 0.2% of the population, respectively.

#### Location of Variants

Twenty-two individuals had a *NOTCH3* EGFR 1-6 variant. Compared with other *NOTCH3* carriers, EGFRs 1-6 carriers had increased risk of migraine (OR, 6.61; 95% CI, 2.14-17.89; *P* = .002), any stroke (OR, 14.01; 95% CI, 5.34-36.56; *P* = 6.6 × 10^−7^), ischemic stroke (OR, 13.78; 95% CI, 4.92-36.73; *P* = 5.7 × 10^−6^), vascular dementia (OR, 82.68; 95% CI, 22.49-358.03; *P* = 4.9 × 10^−10^) and all-cause dementia (OR, 46.19; 95% CI, 14.24-162.62; *P* = 3.0 × 10^−9^) but not of epilepsy (OR, 2.17; 95% CI, 0.23-9.46; *P* = .42) (Table 2).

**Table 2.** Association of Variant Position in *NOTCH3*, *HTRA1*, and *COL4A1/2* With Lifetime Diagnoses of cSVD-Associated Diseases^a^

Medical record detail	Odds ratio (95% CI)		
	*NOTCH3* EGFRs 1-6 vs 7-34 (N_1_ = 22 vs N_2_ = 950)	p.Arg227Trp vs other *HTRA1* variants (N_1_ = 378 vs N_2_ = 167)	COL4A1:p.Gly332Arg vs other *COL4A1/2* (N_1_ = 174 vs N_2_ = 162)
Any stroke	14.01 (5.34-36.56)	0.39 (0.20-0.79)	1.72 (0.61-5.15)
Ischemic stroke	13.78 (4.92-36.73)	0.28 (0.11-0.65)	1.10 (0.23-5.65)
Intracerebral hemorrhage	10.39 (1.78-43.32)	0.59 (0.12-3.38)	5.72 (0.73-102.93)
Family history of stroke	8.55 (3.23-27.99)	1.21 (0.81-1.81)	0.75 (0.45-1.26)
Vascular dementia	82.68 (22.49-358.03)	0.21 (0.03-1.20)	25.46 (0.73-3 144 929.33)
All-cause dementia	46.19 (14.24-162.62)	0.43 (0.16-1.20)	2.74 (0.58-15.87)
Migraine	6.61 (2.14-17.89)	0.92 (0.39-2.35)	2.20 (0.59-11.70)
Migraine with aura	4.31 (0.03-96.82)	0.17 (0.02-0.90)	No cases
Epilepsy	2.17 (0.23-9.46)	0.77 (0.27-2.52)	0.52 (0.06-3.12)

Abbreviations: cSVD, cerebral small vessel disease; EGFRs, epidermal growth factor–like repeats.

^a^ A Firth correction was applied to all the regression models, which were adjusted for age, sex, ethnicity, exome sequencing batch, and the first 10 principal components of genetic ancestry.

The *HTRA1* p.Arg227Trp variant was found in 379 individuals. Compared with other *HTRA1* carriers, p.Arg227Trp carriers had reduced risk of any stroke (OR, 0.39; 95% CI, 0.20-0.79; *P* = .01) and ischemic stroke (OR, 0.28; 95% CI, 0.11-0.65; *P* = .003). They had similar risk of any migraine (OR, 0.92; 95% CI, 0.39-2.35; *P* = .85) but decreased risk of migraine with aura (OR, 0.17; 95% CI, 0.02-0.90; *P* = .04) (Table 2).

The *COL4A1* p.Gly332Arg variant was found in 172 individuals. Compared with other *COL4A1/2* carriers, p.Gly332Arg carriers did not differ in their risk of any of the clinical outcomes assessed (Table 2).

## Discussion

In more than 450 000 individuals, we demonstrated that *NOTCH3*, *HTRA1*, and *COL4A1/2* variants identical to those causing monogenic cSVD are much more frequent than expected in the general population based on the frequency of clinical disease caused by these variants. This is consistent with reports from various population databases,^5,6,36,37^ demonstrating that such variants occur in 2 to 3 individuals per 1000. Our results extend previous work by showing association of such variants with disease. We demonstrated that *NOTCH3* and *HTRA1* variants were associated with increased risk of ischemic stroke and vascular dementia. Although *COL4A1/2* variants were associated with increased risk of any stroke, this was accounted for by a marked increase in intracerebral hemorrhage risk, and no significant difference in ischemic stroke risk was found.

An important question is why many individuals with these variants remain asymptomatic. Our results suggest that cardiovascular risk factors and variant within the gene affect penetrance. For *NOTCH3* and *HTRA1*, cardiovascular risk factors, as assessed by the FRS, increased ischemic stroke risk in variant carriers, and there was a statistical interaction between variant status and FRS. This is consistent with previous cross-sectional data from patients with symptomatic CADASIL in which smoking and hypertension were associated with increased stroke risk.^8,38^ We demonstrated that variant location is associated with disease severity. Previous studies have shown that *NOTCH3* EGFR 1-6 variants are associated with more severe disease,^4,5^ and our data confirmed this with markedly increased stroke and dementia risk in carriers of EGFR 1-6 variants. We extended this finding to show similar effects in *HTRA1* where the p.Arg227Trp variant was found to have lower stroke risk.

In contrast, genetic propensity to common ischemic stroke, as assessed by PRS, was only associated with increased risk in individuals without *NOTCH3* or *HTRA1* variants. It is possible that the PRS may mask variant-level epistasis with constituting variants and/or other common yet unidentified genetic variants. Common genetic variants have been shown to affect the penetrance of monogenic conditions.^39^ In addition, in symptomatic CADASIL cases, WMH volume was demonstrated to have significant heritability after controlling for *NOTCH3* variants, suggesting the existence of modifier genes.^9^

Our MRI analysis showed that *NOTCH3* and *HTRA1* variants were associated with increased WMH volume, reduced white matter integrity as assessed by PSMD, and disruption of structural brain networks as assessed by local and global efficiency. Structural network measures have been shown to mediate the effect of other cSVD pathologies (eg, WMH and lacunar infarcts) on cognition.^28^ Surprisingly, *NOTCH3* variants were associated with increased brain volume. The reason for this is unclear because patients with severe CADASIL have reduced brain volume. It is possible that pathological changes in earlier disease stages are associated with inflammatory or edematous changes that could increase brain volume, although further research is required.

### Strengths and Limitations

Strengths of our study include that we used the largest cohort to date and extended previous work to analyze associations with all 3 common monogenic cSVDs. Furthermore, the prospective nature of UK Biobank allowed us to compare risk of incident stroke conferred by variants to that from a risk prediction score used in clinics.

Our study also has limitations. The study sample was large but not necessarily representative of the wider UK population, and frequency of monogenic stroke variants may differ between ethnic groups, although this should not affect inferences in this study. Second, we were unable to examine associations of variants directly with lacunar stroke because these data were not available. Also, diseases like dementia can sometimes be misclassified or underrepresented by the codes in health records.

## Conclusions

This cohort study demonstrated in more than 450 000 individuals that relatively common variants in genes causing monogenic cSVD are associated with increased risk of both stroke and dementia. This increase in risk is primarily via ischemic stroke for *NOTCH3* and *HTRA1* and via intracerebral hemorrhage for *COL4A1/2*. Our results show that factors contributing to the variation in penetrance include variant location within the gene and conventional vascular risk factors. Our results imply that intensive vascular risk factor control is likely to improve disease prognosis in individuals with these genetic variants and that identifying individuals early before onset of symptoms and covert MRI changes of cSVD might help reduce stroke and dementia risk.

## Notes

Supplement. **eTable 1.** The code list used for the retrieval of disease records **eTable 2.** An overview of the brain MRI scanner and its sequence parameters **eTable 3.** Information about the 99 distinct cysteine-altering *NOTCH3* variants **eTable 4. **Information about the 18 *HTRA1* pathogenic variants found in UK Biobank **eTable 5. **Information about the 11 *COL4A1/2* pathogenic variants found in UK Biobank **eTable 6. **Association of *NOTCH3 *variants with cerebral small vessel disease related diagnoses in unrelated individuals with adjustment for Framingham cardiovascular risk and polygenic risk scores **eTable 7. **Association of *HTRA1 *variants with cerebral small vessel disease related diagnoses in unrelated individuals with adjustment for Framingham cardiovascular risk and polygenic risk scores **eTable 8. **Association of *COL4A1/2 *variants with cerebral small vessel disease related diagnoses in unrelated individuals with adjustment for Framingham cardiovascular risk and polygenic risk scores **eFigure 1. **Flow chart of literature search and selection of studies for *HTRA1* and *COL4A1/2* variants **eFigure 2. **Lolliplot showing the distribution of distinct pathogenic variants in UK Biobank across the reverse strand of the *NOTCH3* gene **eFigure 3. **Lolliplot showing the distribution of distinct pathogenic variants in UK Biobank across the forward strand of the *HTRA1* gene **eFigure 4. **Lolliplot showing the distribution of distinct pathogenic variants in UK Biobank across the reverse strand of the *COL4A1* gene **eFigure 5. **Lolliplot showing the distribution of distinct pathogenic variants in UK Biobank across the forward strand of the *COL4A2* gene
