# Supplementary material for: Association of Vascular Risk Factors and Genetic Factors With Penetrance of Variants Causing Monogenic Stroke
Source: JAMA Neurol. 2022 Oct 27;79(12):1303–11. doi: 10.1001/jamaneurol.2022.3832 (PMC9614680; doi:10.1001/jamaneurol.2022.3832)
Supplement: Supplement. — eTable 1. The code list used for the retrieval of disease records eTable 2. An overview of the brain MRI scanner and its sequence parameters eTable 3. Information about the 99 distinct cysteine-altering NOTCH3 variants eTable 4. Information about the 18 HTRA1 pathogenic variants found in UK Biobank eTable 5. Information about the 11 COL4A1/2 pathogenic variants found in UK Biobank eTable 6. Association of NOTCH3 variants with cerebral small vessel disease related diagnoses in unrelated individuals with adjustment for Framingham cardiovascular risk and polygenic risk scores eTable 7. Association of HTRA1 variants with cerebral small vessel disease related diagnoses in unrelated individuals with adjustment for Framingham cardiovascular risk and polygenic risk scores eTable 8. Association of COL4A1/2 variants with cerebral small vessel disease related diagnoses in unrelated individuals with adjustment for Framingham cardiovascular risk and polygenic risk scores eFigure 1. Flow chart of literature search and selection of studies for HTRA1 and COL4A1/2 variants eFigure 2. Lolliplot showing the distribution of distinct pathogenic variants in UK Biobank across the reverse strand of the NOTCH3 gene eFigure 3. Lolliplot showing the distribution of distinct pathogenic variants in UK Biobank across the forward strand of the HTRA1 gene eFigure 4. Lolliplot showing the distribution of distinct pathogenic variants in UK Biobank across the reverse strand of the COL4A1 gene eFigure 5. Lolliplot showing the distribution of distinct pathogenic variants in UK Biobank across the forward strand of the COL4A2 gene [file jamaneurol-e223832-s001.pdf]

Supplemental Online Content

Cho BPH, Harshfield EL, Al-Thani M, Tozer DJ, Bell S, Markus HS. Association of vascular risk factors and genetic factors with penetrance of variants causing monogenic stroke. *JAMA Neurol*. Published online October 27, 2022. doi:10.1001/jamaneurol.2022.3832

eTable 1. The code list used for the retrieval of disease records
eTable 2. An overview of the brain MRI scanner and its sequence parameters
eTable 3. Information about the 99 distinct cysteine-altering *NOTCH3* variants
eTable 4. Information about the 18 *HTRA1* pathogenic variants found in UK Biobank
eTable 4 references
eTable 5. Information about the 11 *COL4A1/2* pathogenic variants found in UK Biobank
eTable 5 references
eTable 6. Association of *NOTCH3* variants with cerebral small vessel disease related diagnoses in unrelated individuals with adjustment for Framingham cardiovascular risk and polygenic risk scores
eTable 7. Association of *HTRA1* variants with cerebral small vessel disease related diagnoses in unrelated individuals with adjustment for Framingham cardiovascular risk and polygenic risk scores
eTable 8. Association of *COL4A1/2* variants with cerebral small vessel disease related diagnoses in unrelated individuals with adjustment for Framingham cardiovascular risk and polygenic risk scores
eFigure 1. Flow chart of literature search and selection of studies for *HTRA1* and *COL4A1/2* variants
eFigure 2. Lolliplot showing the distribution of distinct pathogenic variants in UK Biobank across the reverse strand of the *NOTCH3* gene
eFigure 3. Lolliplot showing the distribution of distinct pathogenic variants in UK Biobank across the forward strand of the *HTRA1* gene
eFigure 4. Lolliplot showing the distribution of distinct pathogenic variants in UK Biobank across the reverse strand of the *COL4A1* gene
eFigure 5. Lolliplot showing the distribution of distinct pathogenic variants in UK Biobank across the forward strand of the *COL4A2* gene

This supplemental material has been provided by the authors to give readers additional information about their work.

© 2022 Cho BPH et al. *JAMA Neurology.*

eTable 1. The code list used for the retrieval of disease records.

Biobank Code Text	Code type	Code
Migraine	UK Biobank algorithmically defined migraine	131053
	UK Biobank self-report	Field 20002 Code 1265
	ICD9 for hospital record retrieval	346
	ICD10 for hospital record retrieval	G43
Migraine with aura	ICD9 for hospital record retrieval	3460
	ICD10 for hospital record retrieval	G431
Stroke	UK Biobank algorithmically defined stroke	42007
	UK Biobank self-report	Field 20002 Codes 1081,1086,1491,1583
	ICD9 for hospital and death record retrieval	430, 431, 434, 436
	ICD10 for hospital and death record retrieval	I60, I61, I63, I64
Date of stroke	UK Biobank algorithmically defined stroke	42006
	ICD9 for hospital and death record retrieval	430, 431, 434, 436
	ICD10 for hospital and death record retrieval	I60, I61, I63, I64
Ischemic stroke	UK Biobank algorithmically defined ischaemic stroke	42009
	UK Biobank self-report	Field 20002 Code 1583
	ICD9 for hospital and death record retrieval	434, 436
	ICD10 for hospital and death record retrieval	I63, I64
Date of ischemic stroke	UK Biobank algorithmically defined ischaemic stroke	42008
	ICD9 for hospital and death record retrieval	434, 436
	ICD10 for hospital and death record retrieval	I63, I64
Intracerebral hemorrhage (ICH)	UK Biobank algorithmically defined ICH	42011
	UK Biobank self-report	Field 20002 Codes 1491
	ICD9 for hospital and death record retrieval	431
	ICD10 for hospital and death record retrieval	I61
Date of ICH	UK Biobank algorithmically defined ICH	42010
	ICD9 for hospital and death record retrieval	431
	ICD10 for hospital and death record retrieval	I61
Family history of stroke	Illness of father	Field 20107 Code 2
	Illness of mother	Field 20110 Code 2
Vascular dementia	UK Biobank algorithmically defined vascular dementia	42023
	ICD9 for hospital and death record retrieval	290.2,2903,2904,2912,2941,3310,3311,3312,3315
	ICD10 for hospital and death record retrieval	F00, F01, F02, F03, G30, A81.0, F05.1,F10.6,G31.0,G31.1,G31.8,I67.3
Date of vascular dementia	ICD9 for hospital and death record retrieval	290.2,2903,2904,2912,2941,3310,3311,3312,3315
	ICD10 for hospital and death record retrieval	F00, F01, F02, F03, G30, A81.0, F05.1,F10.6,G31.0,G31.1,G31.8,I67.3
All-cause dementia	UK Biobank algorithmically defined dementia	42019
Epilepsy	UK Biobank algorithmically defined epilepsy	131049
	UK Biobank self-report	Field 20002 Code 1264
	ICD9 for hospital and death record retrieval	345
	ICD10 for hospital and death record retrieval	G40

© 2022 Cho BPH et al. *JAMA Neurology.*

eTable 2. An overview of the brain MRI scanner and its sequence parameters.

**Scanner**	**Siemens Skyra 3T**
**T1-weighted structural imaging**	**Straight sagittal orientation** (i.e. with the field-of-view aligned to the scanner axes)<br>**TR** (repetition time)=2000 ms; **TE** (echo time)=2.01 ms<br>**Resolution**:1x1x1 mm; **Field-of-view**: 208x256x256 matrix<br>**Duration**: 5 minutes<br>**Others:** 3D magnetization-prepared rapid acquisition with gradient echo (MPRAGE), in-plane acceleration iPAT=2, prescan-normalise
**T2-weighted FLAIR structural imaging**	**Straight sagittal orientation**<br>**TR**=5000 ms; **TE**=395 ms<br>**Resolution**: 1.05x1x1 mm; **Field-of-view**: 192x256x256 matrix;<br>**Duration**: 6 minutes<br>**Others**: 3D sampling perfection with application-optimized contrasts by using flip angle evolution (SPACE), in-plane acceleration integrated Parallel Acquisition Techniques (iPAT)=2, partial Fourier = 7/8, fat saturation, elliptical k-space scanning, prescan-normalise
**Diffusion imaging**	**TR**=3600 ms; **TE**=92 ms<br>**Resolution**: 2x2x2 mm; **Field-of-view**: 104x104x72 matrix<br>**Duration**: 7 minutes (including 36 seconds phase-encoding reversed data), 5x b=0 (+3x b=0 blip-reversed), 50x b=1000 s/mm^2, 50x b=2000 s/mm^2<br>**Gradient timings**: duration=21.4 ms, spacing = 45.5 ms; Spoiler b-value = 3.3 s/mm^2<br>**Number of gradient directions:** For the two diffusion-weighted shells, 50 distinct diffusion-encoding directions were acquired (and all 100 directions are distinct). The diffusion preparation is a standard (“monopolar”) Stejskal-Tanner pulse sequence.<br>**Others:** Spin-echo echo planar imaging (SE-EPI) with x3 multislice acceleration, no iPAT, fat saturation

© 2022 Cho BPH et al. *JAMA Neurology.*

eTable 3. Information about the 99 distinct cysteine-altering *NOTCH3* variants.

Protein change	Genomic change	Codon change	Exon	EGFR domain	Number of carriers in the UK Biobank	Ethnicities of variant carriers in UK Biobank
p.Cys1372Trp	chr19:g.15177812G>C	c.4116C>G	24	34	1	White (1)
p.Arg1360Cys	chr19:g.15177850G>A	c.4078C>T	24	34	1	White (1)
p.Cys1324Tyr	chr19:g.15177957C>T	c.3971G>A	24	33	1	White (1)
p.Cys1324Ser	chr19:g.15177958A>T	c.3970T>A	24	33	2	White (2)
p.Cys1315Trp	chr19:g.15177983G>C	c.3945C>G	24	33	1	White (1)
p.Cys1315Phe	chr19:g.15177984C>A	c.3944G>T	24	33	10	White (10)
p.Cys1313Ser	chr19:g.15177990C>G	c.3938G>C	24	33	1	White (1)
p.Cys1293Phe	chr19:g.15178050C>A	c.3878G>T	24	33	1	White (1)
p.Arg1291Cys	chr19:g.15178057G>A	c.3871C>T	24	33	2	White (2)
p.Cys1275Ser	chr19:g.15178836C>G	c.3824G>C	23	32	1	White (1)
p.Arg1262Cys	chr19:g.15178876G>A	c.3784C>T	23	32	1	Black or Black British (1)
p.Cys1261Trp	chr19:g.15178877G>C	c.3783C>G	23	32	1	White (1)
p.Cys1250Arg	chr19:g.15178912A>G	c.3748T>C	23	32	1	Asian or Asian British (1)
p.Arg1242Cys	chr19:g.15178936G>A	c.3724C>T	23	31	9	White (9)
p.Arg1231Cys	chr19:g.15179052G>A	c.3691C>T	22	31	255	Asian or Asian British (74), Black or Black British (1), Mixed (6), White (160), Unknown (14)
p.Cys1222Gly	chr19:g.15179079A>C	c.3664T>G	22	31	212	White (212)
p.Cys1222Arg	chr19:g.15179079A>G	c.3664T>C	22	31	1	White (1)
p.Arg1210Cys	chr19:g.15179115G>A	c.3628C>T	22	31	3	White (2), Unknown (1)
p.Arg1201Cys	chr19:g.15179142G>A	c.3601C>T	22	30	52	Black or Black British (1), White (50), Unknown (1)
p.Cys1193Tyr	chr19:g.15179165C>T	c.3578G>A	22	30	1	White (1)
p.Arg1190Cys	chr19:g.15179175G>A	c.3568C>T	22	30	21	Asian or Asian British (1), Black or Black British (1), White (19)
p.Gly1165Cys	chr19:g.15179250C>A	c.3493G>T	22	30	3	White (3)
p.Cys1157Arg	chr19:g.15179274A>G	c.3469T>C	22	29	1	White (1)
p.Tyr1144Cys	chr19:g.15179393T>C	c.3431A>G	21	29	2	White (2)
p.Arg1143Cys	chr19:g.15179397G>A	c.3427C>T	21	29	133	Black or Black British (2), White (130), Unknown(1)
p.Cys1137Arg	chr19:g.15179415A>G	c.3409T>C	21	29	1	White (1)
p.Cys1119Tyr	chr19:g.15179468C>T	c.3356G>A	21	28	6	White (6)
p.Cys1110Arg	chr19:g.15179496A>G	c.3328T>C	21	28	4	White (4)
p.Cys1108Arg	chr19:g.15180077A>G	c.3322T>C	20	28	2	White (2)
p.Arg1100Cys	chr19:g.15180101G>A	c.3298C>T	20	28	3	Black or Black British (1), White (2)
p.Arg1076Cys	chr19:g.15180173G>A	c.3226C>T	20	27	1	White (1)
p.Cys1061Tyr	chr19:g.15180217C>T	c.3182G>A	20	27	3	White (3)
p.Cys1055Tyr	chr19:g.15180235C>T	c.3164G>A	20	27	1	White (1)

© 2022 Cho BPH et al. *JAMA Neurology.*

p.Arg1031Cys	chr19:g.15180732G>A	c.3091C>T	19	26	3	White (3)
p.Cys1015Arg	chr19:g.15180780A>G	c.3043T>C	19	26	1	White (1)
p.Arg1006Cys	chr19:g.15180807G>A	c.3016C>T	19	26	1	White (1)
p.Cys1004Tyr	chr19:g.15180812C>T	c.3011G>A	19	26	1	White (1)
p.Cys986Gly	chr19:g.15180999A>C	c.2956T>G	18	25	1	Chinese (1)
p.Cys986Arg	chr19:g.15180999A>G	c.2956T>C	18	25	1	White (1)
p.Cys971Tyr	chr19:g.15181043C>T	c.2912G>A	18	25	1	White (1)
p.Cys948Phe	chr19:g.15181112C>A	c.2843G>T	18	24	1	Black or Black British (1)
p.Cys939Ser	chr19:g.15181139C>G	c.2816G>C	18	24	2	White (1), Unknown(1)
p.Tyr916Cys	chr19:g.15181621T>C	c.2747A>G	17	23	10	White (10)
p.Cys912Ser	chr19:g.15181633C>G	c.2735G>C	17	23	2	White (2)
p.Cys910Tyr	chr19:g.15181639C>T	c.2729G>A	17	23	5	White (5)
p.Cys910Arg	chr19:g.15181640A>G	c.2728T>C	17	23	2	White (2)
p.Cys873Arg	chr19:g.15181751A>G	c.2617T>C	17	22	1	White (1)
p.Gly861Cys	chr19:g.15181787C>A	c.2581G>T	17	22	1	Black or Black British (1)
p.Trp802Cys	chr19:g.15184910C>A	c.2406G>T	15	20	6	White (6)
p.Cys798Tyr	chr19:g.15184923C>T	c.2393G>A	15	20	2	White (2)
p.Cys796Ser	chr19:g.15184929C>G	c.2387G>C	15	20	1	White (1)
p.Arg785Cys	chr19:g.15184963G>A	c.2353C>T	15	20	12	White (12)
p.Arg767Cys	chr19:g.15185017G>A	c.2299C>T	15	19	12	Chinese (1), White (10), Mixed (1)
p.Ser740Cys	chr19:g.15185334G>C	c.2219C>G	14	19	1	White (1)
p.Arg728Cys	chr19:g.15185371G>A	c.2182C>T	14	18	15	White (13), Unknown(2)
p.Arg717Cys	chr19:g.15185404G>A	c.2149C>T	14	18	29	White (29)
p.Tyr710Cys	chr19:g.15185502T>C	c.2129A>G	13	18	1	Chinese (1)
p.Arg680Cys	chr19:g.15185593G>A	c.2038C>T	13	17	3	Asian or Asian British (1), White (2)
p.Cys672Tyr	chr19:g.15185616C>T	c.2015G>A	13	17	1	Unknown(1)
p.Ser671Cys	chr19:g.15185619G>C	c.2012C>G	13	17	4	White (4)
p.Gly667Cys	chr19:g.15185632C>A	c.1999G>T	13	17	2	White (1), Unknown(1)
p.Cys654Tyr	chr19:g.15185670C>T	c.1961G>A	13	16	1	White (1)
p.Arg640Cys	chr19:g.15186911G>A	c.1918C>T	12	16	20	Asian or Asian British (1), Black or Black British (1), Chinese (1), White (14), Unknown (3)
p.Cys629Arg	chr19:g.15186944A>G	c.1885T>C	12	16	1	White (1)
p.Cys608Trp	chr19:g.15187121G>C	c.1824C>G	11	15	1	White (1)
p.Arg607Cys	chr19:g.15187126G>A	c.1819C>T	11	15	7	White (7)
p.Arg592Cys	chr19:g.15187171G>A	c.1774C>T	11	15	1	White (1)
p.Arg587Cys	chr19:g.15187186G>A	c.1759C>T	11	15	7	Black or Black British (1), Chinese (1), White (4), Unknown (1)
p.Cys579Tyr	chr19:g.15187209C>T	c.1736G>A	11	14	2	Black or Black British (1), White (1)

© 2022 Cho BPH et al. *JAMA Neurology.*

p.Arg578Cys	chr19:g.15187213G>A	c.1732C>T	11	14	13	Asian or Asian British (1), White (12)
p.Arg558Cys	chr19:g.15187273G>A	c.1672C>T	11	14	4	White (4)
p.Cys554Phe	chr19:g.15187284C>A	c.1661G>T	11	14	1	White (1)
p.Arg532Cys	chr19:g.15187893G>A	c.1594C>T	10	13	4	White (4)
p.Cys522Ser	chr19:g.15187922C>G	c.1565G>C	10	13	1	White (1)
p.Cys516Phe	chr19:g.15187940C>A	c.1547G>T	10	13	7	White (7)
p.Cys516Tyr	chr19:g.15187940C>T	c.1547G>A	10	13	1	White (1)
p.Cys511Phe	chr19:g.15187955C>A	c.1532G>T	10	13	1	White (1)
p.Cys504Arg	chr19:g.15187977A>G	c.1510T>C	10	12	1	White (1)
p.Gly481Cys	chr19:g.15188286C>A	c.1441G>T	9	12	2	White (2)
p.Ser476Cys	chr19:g.15188301T>A	c.1426A>T	9	12	3	White (3)
p.Tyr465Cys	chr19:g.15188333T>C	c.1394A>G	9	11	1	White (1)
p.Cys455Tyr	chr19:g.15189003C>T	c.1364G>A	8	11	1	White (1)
p.Arg421Cys	chr19:g.15189106G>A	c.1261C>T	8	10	2	White (2)
p.Cys419Ser	chr19:g.15189112A>T	c.1255T>A	8	10	1	White (1)
p.Cys408Arg	chr19:g.15189145A>G	c.1222T>C	8	10	1	White (1)
p.Cys360Tyr	chr19:g.15189386C>T	c.1079G>A	7	9	1	White (1)
p.Tyr337Cys	chr19:g.15191450T>C	c.1010A>G	6	8	1	White (1)
p.Arg332Cys	chr19:g.15191466G>A	c.994C>T	6	8	1	White (1)
p.Cys318Phe	chr19:g.15191507C>A	c.953G>T	6	8	2	White (2)
p.Cys311Ser	chr19:g.15191529A>T	c.931T>A	6	7	2	White (2)
p.Tyr258Cys	chr19:g.15191774T>C	c.773A>G	5	6	1	Unknown(2)
p.Arg207Cys	chr19:g.15192020G>A	c.619C>T	4	5	2	White (2)
p.Arg182Cys	chr19:g.15192095G>A	c.544C>T	4	4	7	White (7)
p.Arg169Cys	chr19:g.15192134G>A	c.505C>T	4	4	2	White (2)
p.Arg153Cys	chr19:g.15192182G>A	c.457C>T	4	3	1	White (1)
p.Arg141Cys	chr19:g.15192218G>A	c.421C>T	4	3	5	White (5)
p.Arg110Cys	chr19:g.15192389G>A	c.328C>T	3	2	2	White (2)
p.Arg90Cys	chr19:g.15192449G>A	c.268C>T	3	2	1	White (1)
p.Arg54Cys	chr19:g.15197537G>A	c.160C>T	2	1	1	White (1)

© 2022 Cho BPH et al. *JAMA Neurology.*

eTable 4. Information about the 18 *HTRA1* pathogenic variants found in UK Biobank. Details of the ACMG classification are also included. For more details about the ACMG criteria, please refer to references 11 and 12 in the main text.

Protein change	Genomic change	Codon change	Exon	Domain	Frequency in UK Biobank	PVS1	PS1	PS2	PS3	PS4	PP1	PM1	PM2	PM3	PM4	PM5	PM6	PP1_Moderate	PS1_Moderate	PP1	PP2	PP3	PP4	PP5	PM5	ACMG class	Ethnicity of variant carriers in UK Biobank
p.Cys41Ter	g.122461775:C>A	c.123C>A		IGFBP	1					Yes			Yes		Yes											Likely Pathogenic	White (1)
p.Gly120Asp	g.122462011:G>A	c.359G>A	1	KM	1				Yes	Yes			Yes													Pathogenic	White (1)
p.Arg166Cys	g.122488925:C>T	c.496C>T	2		13				Yes		Yes		Yes			Yes						Yes				Pathogenic	White (13)
p.Ala173Thr	g.122488946:G>A	c.517G>A	2		3				Yes				Yes			Yes						Yes				Likely Pathogenic	White (3)
p.Val175Met	g.122488952:G>A	c.523G>A	2		4					Yes			Yes									Yes				Likely Pathogenic	White (4)
p.Arg227Trp	g.122489528:C>T	c.679C>T	3	Trypsin	379				Yes				Yes									Yes				Likely Pathogenic	Black or Black British (1), White (377), Unknown (1)
p.Ala252Thr	g.122489603:G>A	c.754G>A	3	Trypsin	2				Yes	Yes			Yes									Yes				Pathogenic	White (2)
p.Ile256Thr	g.122489616:T>C	c.767T>C	3	Trypsin	13				Yes	Yes			Yes									Yes				Pathogenic	Black or Black British (1), White (12)
p.Arg274Gln	g.122506734:G>A	c.821G>A	4	Trypsin	3				Yes	Yes			Yes									Yes				Pathogenic	White (3)
p.Pro275Leu	g.122506737:C>T	c.824C>T	4	Trypsin	7				Yes		Yes		Yes									Yes				Pathogenic	Mixed (1), White (6)
p.Gly283Arg	g.122506760:G>C	c.847G>A	4	Trypsin	1				Yes				Yes			Yes						Yes				Likely Pathogenic	White (1)
p.Gly295Arg	g.122506796:G>A	c.883G>A	4	Trypsin	7				Yes		Yes		Yes									Yes	Yes			Pathogenic	White (7)
p.Val297Met	g.122506802:G>A	c.889G>A	4	Trypsin	1				Yes	Yes			Yes							Yes		Yes				Pathogenic	White (1)
p.Arg302Ter	g.122506817:C>T	c.904C>T		Trypsin	7				Yes	Yes			Yes		Yes					Yes						Pathogenic	White (6), Unknown (1)
p.Met314Val	g.122506853:A>G	c.940A>G	4	Trypsin	20				Yes				Yes									Yes				Likely Pathogenic	Mixed (1), White (19)
p.Asp320Asn	g.122506871:G>A	c.958G>A	4	Trypsin	42				Yes				Yes	Yes								Yes				Likely Pathogenic	White (42)
p.Ala321Thr	g.122506874:G>A	c.961G>A	4	Trypsin	18				Yes				Yes	Yes								Yes				Likely Pathogenic	White (17), Unknown (1)
p.Arg370Ter	g.122508758:C>T	c.1108C>T		PDZ	24				Yes	Yes			Yes		Yes											Pathogenic	White (24)

© 2022 Cho BPH et al. *JAMA Neurology.*

eTable 4 references

Protein change	Article title	Year	Journal	Volume	Issue	Pages	Authors	DOI
p.Cys41Ter	Heterozygous HTRA1 nonsense or frameshift mutations are pathogenic.	2021	Brain	144	9	2616-2624	Coste T, Hervé D, Neau JP et al.	10.1093/brain/awab271
p.Gly120Asp	Characterization of Heterozygous HTRA1 Mutations in Taiwanese Patients With Cerebral Small Vessel Disease	2018	Stroke	49	7	1593-1601	Lee YC, Chung CP, Chao NC et al.	10.1161/STROKEAHA.118.021283
p.Arg166Cys	The first Greek case of heterozygous cerebral autosomal recessive arteriopathy with subcortical infarcts and leukoencephalopathy: An atypical clinico-radiological presentation.	2017	The neuroradiology journal	30	6	583-585	Bougea A, Velonakis G, Spantideas N et al.	10.1177/1971400917700168
	A new Italian family with HTRA1 mutation associated with autosomal-dominant variant of CARASIL: Are we pointing towards a disease spectrum?	2019	Journal of the neurological sciences	396		108-111	Favaretto S, Margoni M, Salviati L et al.	10.1016/j.jns.2018.11.008
	Shifting the CARASIL paradigm: report of a non-Asian family and literature review.	2015	Stroke	46	4	1110-2	Cordeiro IM, Nzwalo I, Sá F et al.	10.1161/STROKEAHA.114.006735
	A Japanese family of autosomal dominant cerebral small vessel disease with heterozygous HTRA1 mutation showing dementia, gait disturbance and subarachnoid hemorrhage	2018	Vas-Cog Journal			20-20	Yamashita T, Nozaki H, Wakutani Y et al.	
	HTRA1-related autosomal dominant cerebral small vessel disease.	2020	Chinese medical journal	134	2	178-184	Liu JY, Zhu YC, Zhou LX et al.	10.1097/CM9.0000000000001176
p.Ala173Thr	A novel HTRA1 exon 2 mutation causes loss of protease activity in a Pakistani CARASIL patient.	2015	Journal of neurology	262	5	1369-1372	Khaleeli Z, Jaunmuktane Z, Beaufort N et al.	10.1007/s00415-015-7769-5
p.Val175Met	New Mutations Linked to Cerebral Autosomal Recessive Arteriopathy With Subcortical Infarcts and Leukoencephalopathy in Africa and North America	2020	Stroke	51		A38-A38	Olowu A and Septien S, Khera A et al.	10.1161/str.51.suppl_1.38
	Heterozygous mutations of HTRA1 gene in patients with familial cerebral small vessel disease.	2017	CNS neuroscience & therapeutics	23	9	759-765	Di Donato I, Bianchi S, Gallus GN et al.	10.1111/cns.12722
	HTRA1-related autosomal dominant cerebral small vessel disease.	2020	Chinese medical journal	134	2	178-184	Liu JY, Zhu YC, Zhou LX et al.	10.1097/CM9.0000000000001176
	Two Unique Mutations in HTRA1-Related Cerebral Small Vessel Disease in North America and Africa and Literature Review.	2021	Journal of stroke and cerebrovascular disease	30	11	106029	Shang T, Pinho M, Ray D et al	10.1016/j.jstrokecerebrovasdis.2021.106029
	Genotype-phenotype correlations of heterozygous HTRA1-related cerebral small vessel disease: case report and systematic review.	2021	Neurogenetics	22	3	187-194	Zhang H, Qin X, Shi Y et al.	10.1007/s10048-021-00646-5
	Clinicoradiographic and genetic features of cerebral small vessel disease indicate variability in mode of inheritance for monoallelic HTRA1 variants.	2021	Molecular genetics & genomic medicine			e1799	Muthusamy K, Ferrer A, Klee EW et al.	10.1002/mgg3.1799
p.Arg227Trp	How common are single gene mutations as a cause for lacunar stroke? A targeted gene panel study.	2019	Neurology	93	22	e2007-e2020	Tan RYY, Traylor M, Megy K et al.	10.1212/WNL.0000000000008544
p.Ala252Thr	Association of HTRA1 mutations and familial ischemic cerebral small-vessel disease.	2009	The New England journal of medicine	360	17	1729-1739	Hara K, Shiga A, Fukutake T et al.	10.1056/NEJMoa0801560
p.Ile256Thr	Characterization of Heterozygous HTRA1 Mutations in Taiwanese Patients With Cerebral Small Vessel Disease.	2018	Stroke	49	7	1593-1601	Lee YC, Chung CP, Chao NC et al.	10.1161/STROKEAHA.118.021283
p.Arg274Gln	A novel mutation in the HTRA1 gene causes CARASIL without alopecia.	2011	Neurology	76	15	1353-1355	Nishimoto Y, Shibata M, Nihonmatsu M et al.	10.1212/WNL.0b013e318215281d
p.Pro275Leu	One Disease with two Faces: Semidominant Inheritance of a Novel HTRA1 Mutation in a Consanguineous Family.	2021	Journal of stroke and cerebrovascular disease	30	9	105997	Bekircan-Kurt CE, Çetinkaya A, Gocmen R et al.	10.1016/j.jstrokecerebrovasdis.2021.105997
	Genotype-phenotype correlations of heterozygous HTRA1-related cerebral small vessel disease: case report and systematic review.	2021	Neurogenetics	22	3	187-194	Zhang H, Qin X, Shi Y et al.	10.1007/s10048-021-00646-5
p.Gly283Arg	Cerebral small vessel disease due to a unique heterozygous HTRA1 mutation in an African man.	2020	Neurology Genetics	6	1	e382-e382	Oluwole OJ, Ibrahim H, Garozzo D et al.	10.1212/NXG.0000000000000382
p.Gly295Arg	A missense HTRA1 mutation expands CARASIL syndrome to the Caucasian population.	2010	Neurology	75	22	2033-2035	Mendioroz M, Fernández-Cadenas I, Del Río-Espinola A et	10.1212/WNL.0b013e3181ff96ac
	Heterozygous mutations of HTRA1 gene in patients with familial cerebral small vessel disease.	2017	CNS neuroscience & therapeutics	23	9	759-765	Di Donato I, Bianchi S, Gallus GN et al.	10.1111/cns.12722
p.Val297Met	Association of HTRA1 mutations and familial ischemic cerebral small-vessel disease.	2009	The New England journal of medicine	360	17	1729-1739	Hara K, Shiga A, Fukutake T et al.	10.1056/NEJMoa0801560
	Clinicoradiographic and genetic features of cerebral small vessel disease indicate variability in mode of inheritance for monoallelic HTRA1 variants.	2021	Molecular genetics & genomic medicine	9	10	e1799	Muthusamy K, Ferrer A, Klee EW et al.	10.1002/mgg3.1799
p.Arg302Ter	Association of HTRA1 mutations and familial ischemic cerebral small-vessel disease.	2009	The New England journal of medicine	360	17	1729-1739	Hara K, Shiga A, Fukutake T et al.	10.1056/NEJMoa0801560
	Unusual case of cerebral small vessel disease with a heterozygous nonsense mutation in HTRA1.	2016	Journal of the neurological sciences	362		144-146	Tateoka T, Onda H, Hirota K et al.	10.1016/j.jns.2016.01.037
	Cerebral Small Vessel Disease Related to a Heterozygous Nonsense Mutation in HTRA1.	2020	Internal medicine	59	10	1309-1313	Ohta K, Ozawa T, Fujinaka H et al.	10.2169/internalmedicine.4041-19
	Heterozygous HTRA1 nonsense or frameshift mutations are pathogenic.	2021	Brain	144	9	2616-2624	Coste T, Hervé D, Neau JP et al.	10.1093/brain/awab271
	Cerebral autosomal recessive arteriopathy with subcortical infarcts and leukoencephalopathy (CARASIL).	2021	Practical neurology	21	5	448-451	Tan RY, Drazyk AM, Urankar K et al.	10.1136/practneurol-2021-003058
	High Diagnostic Utility Incorporating a Targeted Neurodegeneration Gene Panel With MRI Brain Diagnostic Algorithms in Patients With Young-Onset Cognitive Impairment With Leukod	2021	Frontiers in neurology	12		631407	Chen Z, Tan YJ, Lian MM et al.	10.3389/fneur.2021.631407
p.Met314Val	How common are single gene mutations as a cause for lacunar stroke? A targeted gene panel study.	2019	Neurology	93	22	e2007-e2020	Tan RYY, Traylor M, Megy K et al.	10.1212/WNL.0000000000008544
p.Asp320Asn	How common are single gene mutations as a cause for lacunar stroke? A targeted gene panel study.	2019	Neurology	93	22	e2007-e2020	Tan RYY, Traylor M, Megy K et al.	10.1212/WNL.0000000000008544
	A Chinese CARASIL Patient Caused by Novel Compound Heterozygous Mutations in HTRA1.	2018	Journal of stroke and cerebrovascular disease	27	10	2840-2842	Xie F and Zhang LS	10.1016/j.jstrokecerebrovasdis.2018.06.017
	Clinicoradiographic and genetic features of cerebral small vessel disease indicate variability in mode of inheritance for monoallelic HTRA1 variants.	2021	Molecular genetics & genomic medicine	9	10	e1799	Muthusamy K, Ferrer A, Klee EW et al.	10.1002/mgg3.1799
p.Ala321Thr	Whole-exome sequencing of Finnish patients with vascular cognitive impairment.	2021	European journal of human genetics	29	4	663-671	Mönkäre S, Kuuluvainen L, Kun-Rodrigues C et al.	10.1038/s41431-020-00775-9
p.Arg370Ter	Mutation in the HTRA1 gene in a patient with degenerated spine as a component of CARASIL syndrome.	2014	Turkish neurosurgery	24	1	67-69	Bayrakli F, Balaban H, Gurelik M et al.	10.5137/1019-5149.JTN.6226-12.1
	Association of HTRA1 mutations and familial ischemic cerebral small-vessel disease.	2009	The New England journal of medicine	360	17	1729-1739	Hara K, Shiga A, Fukutake T et al.	10.1056/NEJMoa0801560
	Heterozygous HTRA1 nonsense or frameshift mutations are pathogenic.	2021	Brain	144	9	2616-2624	Coste T, Hervé D, Neau JP et al.	10.1093/brain/awab271

© 2022 Cho BPH et al. *JAMA Neurology.*

eTable 5. Information about the 11 *COL4A1/2* pathogenic variants found in UK Biobank. Details of the ACMG classification are also included. For more details about the ACMG criteria, please refer to references 11 and 12 in the main text.

Protein change	Genomic change	Codon change	Exon	Domain	Frequency in UK Biobank	PVS1	PS1	PS2	PS3	PS4	PM1_Strong	PP1_Strong	PM1	PM2	PM3	PM4	PM5	PM6	PP1	PP2	PP3	PP4	PP5	PM3	ACMG class	Ethnicity of variant carriers in UK Biobank
COL4A1:p.Gly1378Asp	g.110164879:C>T	c.4133G>A	46	Triple helix	1					Yes	Yes			Yes							Yes				Pathogenic	White (1)
COL4A1:p.Gly1360Arg	g.110164934:C>T	c.4078G>A	46	Triple helix	2						Yes			Yes							Yes				Likely Pathogenic	Asian or Asian British (1), Unknown (1)
COL4A1:p.Gly1278Ser	g.110169673:C>T	c.3832G>T	43	Triple helix	4					Yes	Yes			Yes							Yes			Yes	Pathogenic	White (4)
COL4A1:p.Arg1063Ter	g.110175229:G>A	c.3187C>T	37	Triple helix	1			Yes			Yes			Yes											Pathogenic	White (1)
COL4A1:p.Gly749Ser	g.110179370:C>T	c.2245G>A	30	Triple helix	2					Yes	Yes			Yes					Yes		Yes				Pathogenic	White (2)
COL4A1:p.Gly720Asp	g.110181326:C>T	c.2159G>A	29	Triple helix	1					Yes	Yes			Yes					Yes		Yes				Pathogenic	White (1)
COL4A1:p.Pro352Leu	g.110201467:G>A	c.1055C>T	19	Triple helix	11				Yes	Yes				Yes							Yes				Pathogenic	Black or Black British (1), White (10)
COL4A1:p.Gly332Arg	g.110203571:C>G	c.994G>C	18	Triple helix	174						Yes			Yes							Yes				Likely Pathogenic	Mixed (1), White (173)
COL4A2:p.Gly466Ser	g.110457399:G>A	c.1396G>A	21	Triple helix	117						Yes			Yes							Yes				Likely Pathogenic	White (117)
COL4A2:p.Gly563Ser	g.110462295:G>A	c.1687G>A	24	Triple helix	3						Yes			Yes							Yes				Likely Pathogenic	White (3)
COL4A2:p.Gly639Ser	g.110465543:G>A	c.1915G>A	25	Triple helix	20						Yes			Yes							Yes				Likely Pathogenic	Asian or Asian British (1), Black or Black British (1), White (17), Unknown (1)

© 2022 Cho BPH et al. *JAMA Neurology.*

eTable 5 references

Protein change	Article title	Year	Journal	Volume	Issue	Pages	Authors	DOI
COL4A1:p.Gly1378Asp	COL4A1 mutations associated with a characteristic pattern of intracranial calcification.	2011	Neuropediatrics	42	6	227-233	Livingston J, Doherty D, Orcesi S et al.	10.1055/s-0031-1295493
COL4A1:p.Gly1360Arg	Prenatal clinical manifestations in individuals with COL4A1/2 variants	2020	Journal of Medical Genetics	58		505-513	Toshiyuki I, Satoko M, Masataka T et al.	http://dx.doi.org/10.1136/jmedgenet-2020-106896
COL4A1:p.Gly1278Ser	COL4A1-related autosomal recessive encephalopathy in 2 Turkish children.	2020	Neurology. Genetics	6	1	e392-e392	Yaramis A, Lochmüller H, Töpf A et al.	10.1212/NXG.0000000000000392
COL4A1:p.Arg1063Ter	Brain and Placental Pathology in Fetal COL4A1 Related Disease.	2021	Pediatric and developmenta	24	3	175-186	Shannon P, Hum C, Parks T et al.	10.1177/1093526620984083
COL4A1:p.Gly749Ser	Suggestive evidence for linkage to chromosome 13qter for autosomal dominant type 1 porencephaly.	2004	Neurology	62	9	1613-5	Aguglia U, Gambardella A, Breedveld GJ et al.	10.1212/01.wnl.0000123113.46672.68
	Mutations in Col4a1 Cause Perinatal Cerebral Hemorrhage and Porencephaly	2005	Science	308	5725	1167-1171	Gould DB, Phalan FC, Breedveld GJ et al.	10.1126/science.1109418
	Fetal origin of brain damage in 2 infants with a COL4A1 mutation: fetal and neonatal MRI.	2011	Neuropediatrics	42	1	1-3	Vermeulen RJ, Peeters-Scholte C, Van Vugt JJ et	10.1055/s-0031-1275343
COL4A1:p.Gly720Asp	COL4A1 mutation in Axenfeld-Rieger anomaly with leukoencephalopathy and stroke.	2007	Annals of neurology	62	2	177-184	Sibon I, Coupry I, Menegon P et al.	10.1002/ana.21191
	COL4A1-related disease: raised creatine kinase and cerebral calcification as useful pointers.	2012	Neuropediatrics	43	5	283-288	Tonduti D, Pichiecchio A, La Piana R et al.	10.1055/s-0032-1325116
	Cerebral small vessel disease with hemorrhagic stroke related to COL4A1 mutation: A case report.	2020	Neuropathology	40	1	93-98	Nandeesh BN, Bindu PS, Narayanappa G et al.	https://pubmed.ncbi.nlm.nih.gov/31808207/
COL4A1:p.Pro352Leu	COL4A1 mutations in patients with sporadic late-onset intracerebral hemorrhage	2012	Annals of neurology	71	4	470-477	Weng YC, Sonni A, Labelle-Dumais C et al.	10.1002/ana.22682
COL4A1:p.Gly332Arg	How common are single gene mutations as a cause for lacunar stroke? A targeted gene panel study.	2019	Neurology	93	22	e2007-e202	Tan RYY, Traylor M, Megy K et al.	10.1212/WNL.0000000000008544
COL4A2:p.Gly466Ser	How common are single gene mutations as a cause for lacunar stroke? A targeted gene panel study.	2019	Neurology	93	22	e2007-e202	Tan RYY, Traylor M, Megy K et al.	10.1212/WNL.0000000000008544
COL4A2:p.Gly563Ser	How common are single gene mutations as a cause for lacunar stroke? A targeted gene panel study.	2019	Neurology	93	22	e2007-e202	Tan RYY, Traylor M, Megy K et al.	10.1212/WNL.0000000000008544
COL4A2:p.Gly639Ser	Fetal origin of brain damage in 2 infants with a COL4A1 mutation: fetal and neonatal MRI.	2011	Neuropediatrics	42	1	1-3	Vermeulen RJ, Peeters-Scholte C, Van Vugt JJ et	10.1055/s-0031-1275343

© 2022 Cho BPH et al. *JAMA Neurology.*

eTable 6. Association of *NOTCH3* variants with cerebral small vessel disease related diagnoses in unrelated individuals with adjustment for Framingham cardiovascular risk and polygenic risk scores (n=379,204, 814 of which were *NOTCH3* carriers).

Medical Records	Number of non-carrier cases	Number of *NOTCH3* cases	Odds ratio [95% CI]	*P*
Any stroke	13665 (3.6%)	62 (7.6%)	2.21 [1.67, 2.87]	2.49E-07
Ischemic stroke	7899 (2.1%)	43 (5.3%)	2.56 [1.81, 3.49]	6.83E-07
Intracerebral hemorrhage	1764 (0.5%)	9 (1.1%)	2.64 [1.29, 4.73]	0.01049165
Family history of stroke	101807 (27.0%)	279 (34.3%)	1.44 [1.24, 1.67]	1.83E-06
Vascular dementia	1280 (0.3%)	14 (1.7%)	5.94 [3.26, 9.91]	5.70E-07
All-cause dementia	5749 (1.5%)	25 (3.1%)	2.36 [1.52, 3.49]	0.0002833
Migraine	15935 (4.2%)	42 (5.2%)	1.35 [0.97, 1.82]	0.0703793
Migraine with aura	377 (0.1%)	1 (0.1%)	1.93 [0.22, 6.91]	0.46882009
Epilepsy	5687 (1.5%)	21 (2.6%)	1.68 [1.03, 2.55]	0.03754576

eTable 7. Association of *HTRA1* variants with cerebral small vessel disease related diagnoses in unrelated individuals with adjustment for Framingham cardiovascular risk and polygenic risk scores (n=379,204, 446 of which were *HTRA1* carriers).

Medical Records	Number of non-carrier cases	Number of *HTRA1* cases	Odds ratio [95% CI]	*P*
Any stroke	13665 (3.6%)	29 (6.5%)	1.86 [1.24, 2.67]	0.00329898
Ischemic stroke	7899 (2.1%)	18 (4.0%)	1.96 [1.18, 3.05]	0.01124191
Intracerebral hemorrhage	1764 (0.5%)	4 (0.9%)	2.13 [0.71, 4.79]	0.15747015
Family history of stroke	101807 (27.0%)	154 (34.5%)	1.38 [1.13, 1.68]	0.00168282
Vascular dementia	1280 (0.3%)	3 (0.7%)	2.21 [0.62, 5.50]	0.19337633
All-cause dementia	5749 (1.5%)	14 (3.1%)	2.01 [1.10, 3.36]	0.02468126
Migraine	15935 (4.2%)	16 (3.6%)	0.84 [0.49, 1.33]	0.47324598
Migraine with aura	377 (0.1%)	4 (0.9%)	10.31 [3.43, 23.38]	0.00035237
Epilepsy	5687 (1.5%)	11 (2.5%)	1.75 [0.92, 3.00]	0.08450765

eTable 8. Association of *COL4A1/2* variants with cerebral small vessel disease related diagnoses in unrelated individuals with adjustment for Framingham cardiovascular risk and polygenic risk scores (n=379,204, 278 of which were *COL4A1/2* carriers).

Medical Records	Number of non-carrier cases	Number of *COL4A1/2* cases	Odds ratio [95% CI]	*P*
Any stroke	13665 (3.6%)	16 (5.8%)	1.54 [0.88, 2.51]	0.12254983
Ischemic stroke	7899 (2.1%)	6 (2.2%)	1.09 [0.45, 2.20]	0.82893632
Intracerebral hemorrhage	1764 (0.5%)	5 (1.8%)	3.52 [1.17, 7.97]	0.02811645
Family history of stroke	101807 (27.0%)	78 (28.1%)	1.09 [0.83, 1.41]	0.53749995
Vascular dementia	1280 (0.3%)	1 (0.4%)	0.52 [0.00, 3.57]	0.60465569
All-cause dementia	5749 (1.5%)	4 (1.4%)	0.85 [0.24, 2.13]	0.76456723
Migraine	15935 (4.2%)	9 (3.2%)	0.86 [0.42, 1.55]	0.63585279
Migraine with aura	377 (0.1%)	0 (0.0%)	1.86 [0.01, 12.74]	0.6904011
Epilepsy	5687 (1.5%)	4 (1.4%)	1.04 [0.35, 2.34]	0.93981102

© 2022 Cho BPH et al. *JAMA Neurology.*

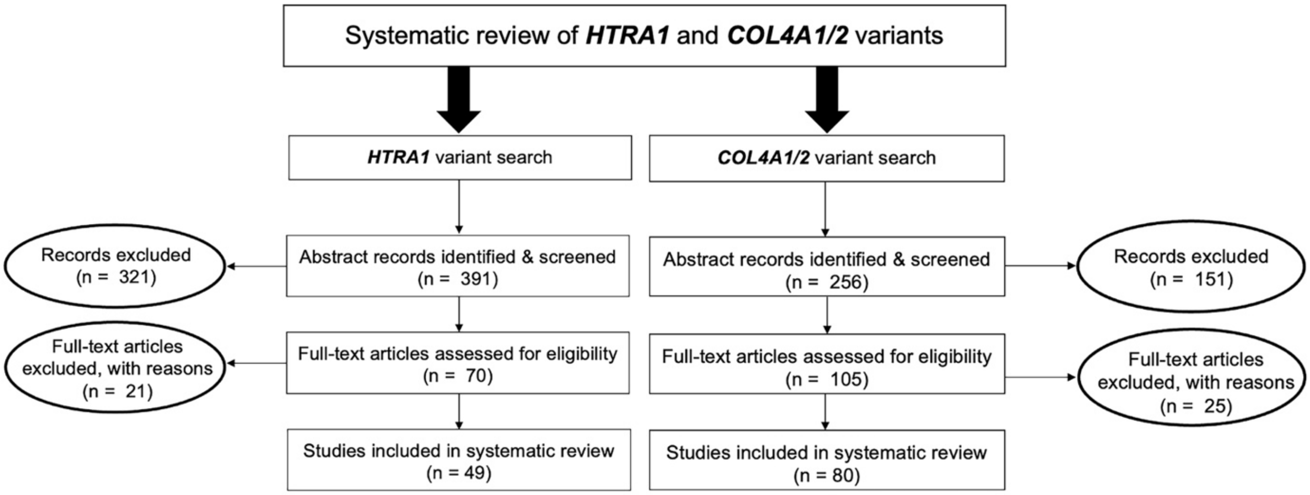

eFigure 1. Flow chart of literature search and selection of studies for *HTRA1* and *COL4A1/2* variants. 77 unique *HTRA1* variants were found in 135 patients, 164 unique *COL4A1* variants in 275 patients, and 34 *COL4A2* variants in 51 patients. PubMed search of English publications until 6-February-2022.

© 2022 Cho BPH et al. *JAMA Neurology.*

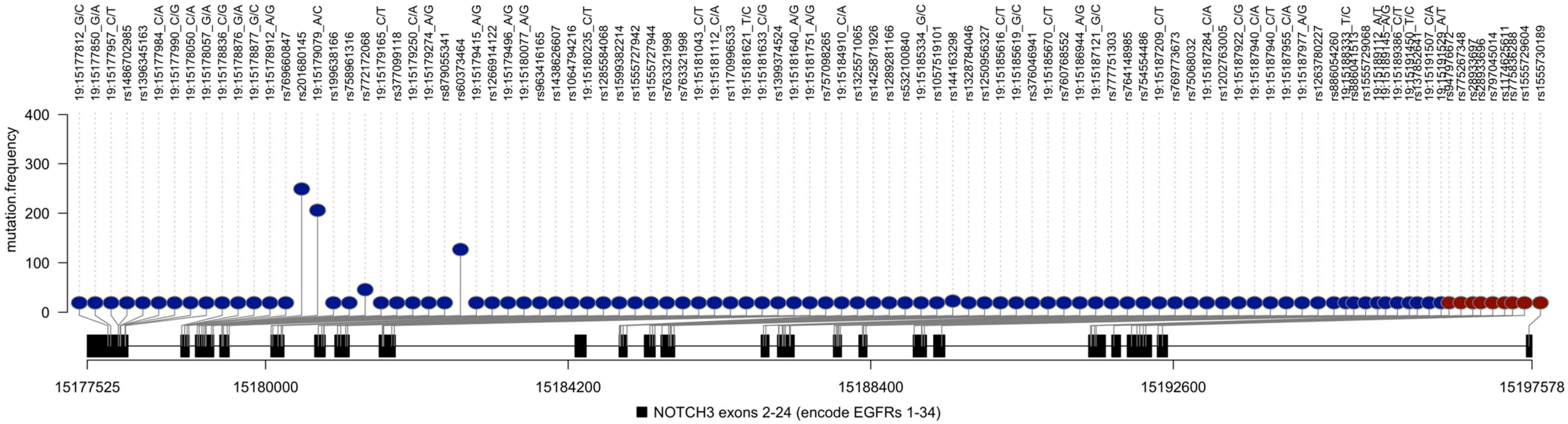

eFigure 2. Lolliplot showing the distribution of distinct pathogenic variants in UK Biobank across the reverse strand of the *NOTCH3* gene. Blue circles represent variants affecting EGFRs 7-34; red circles represent variants affecting EGFRs 1–6. EGFR, epidermal growth factor-like repeat.

© 2022 Cho BPH et al. *JAMA Neurology.*

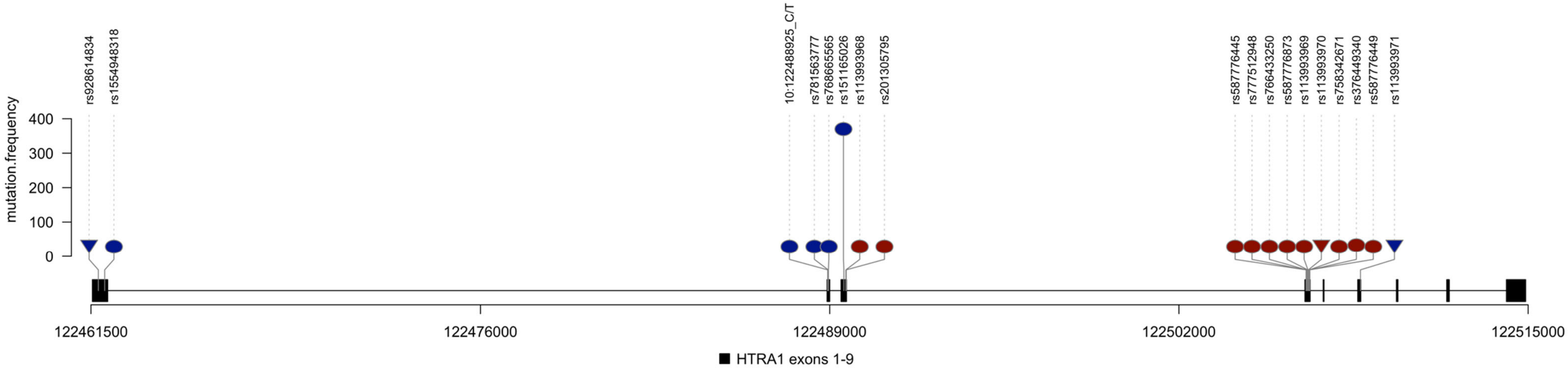

eFigure 3. Lolliplot showing the distribution of distinct pathogenic variants in UK Biobank across the forward strand of the *HTRA1* gene. Red represents variants affecting the protease domain; blue represents other variants; circle represents missense variants; inverted triangle represents a nonsense variant.

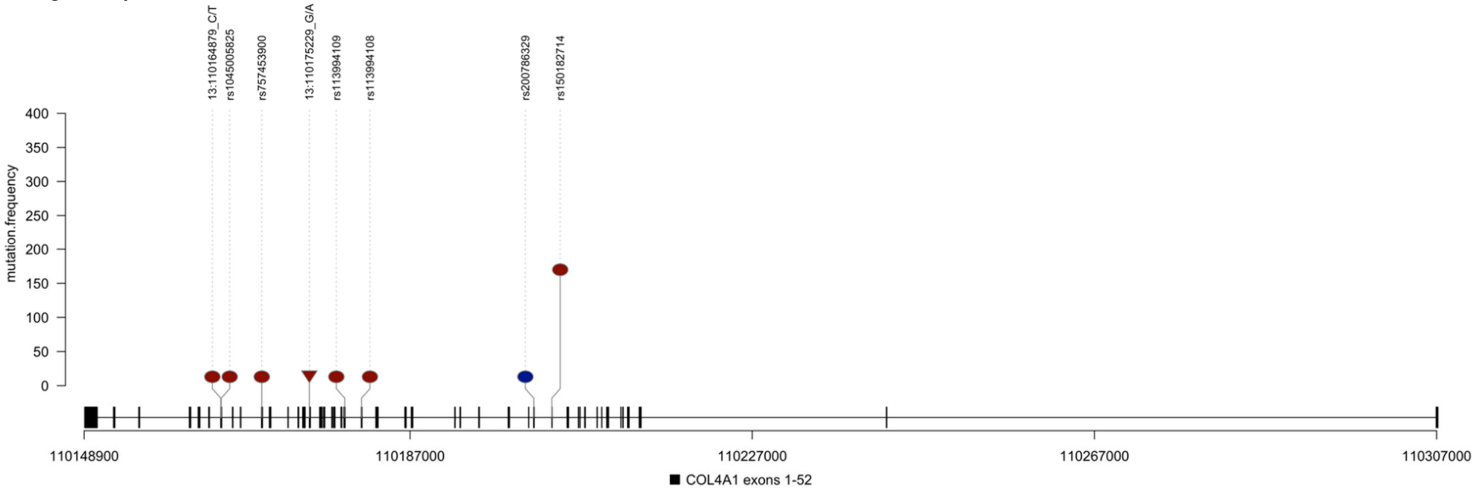

eFigure 4. Lolliplot showing the distribution of distinct pathogenic variants in UK Biobank across the reverse strand of the *COL4A1* gene. Red represents glycine-changing variants affecting the triple helix region; blue represents other variants; circle represents missense variants; inverted triangle represents a nonsense variant.

© 2022 Cho BPH et al. *JAMA Neurology.*

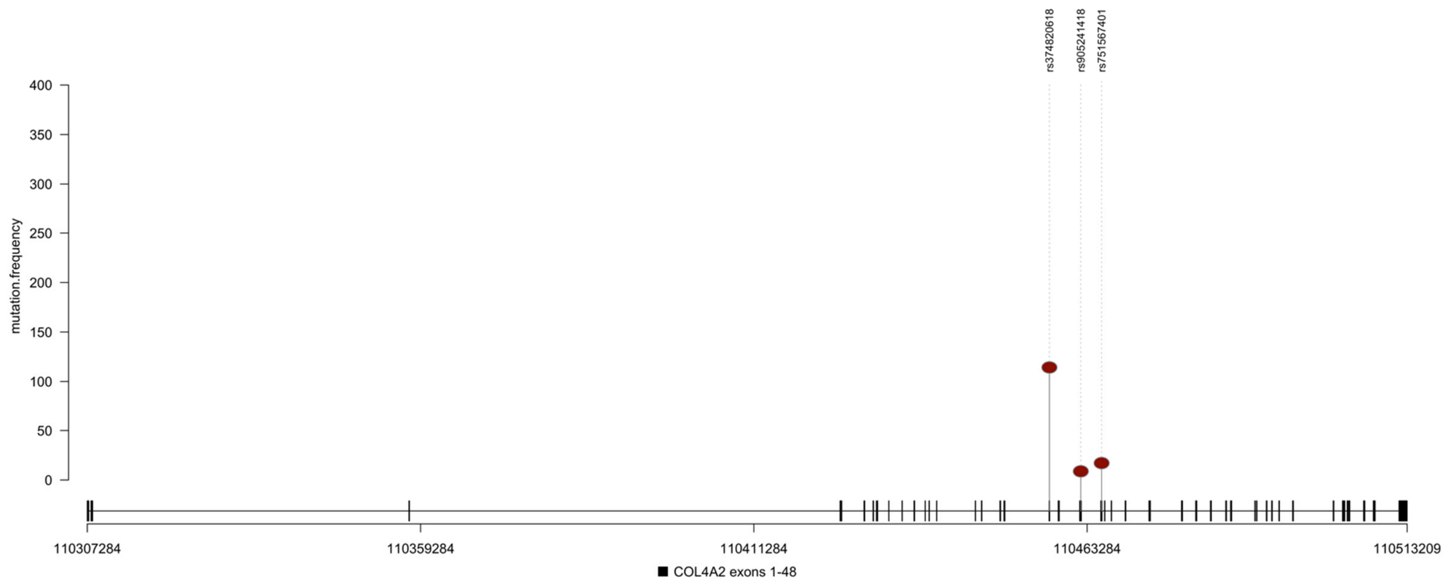

eFigure 5. Lolliplot showing the distribution of distinct pathogenic variants in UK Biobank across the forward strand of the *COL4A2* gene. Red circle represents glycine-changing variants affecting the triple helix region.

© 2022 Cho BPH et al. *JAMA Neurology.*
